# Meclizine and Cephalosporin Combination as Promising Antibiotic Drugs: Formulation, Antimicrobial, and *In Silico* Studies

**DOI:** 10.1155/adpp/8972067

**Published:** 2025-10-14

**Authors:** Wafa M. Al-Madhagi, Mohammed A. Alkhawlani, Ahmed M. Sabati, Arwa Alshargabi, Mohammed Mostafa Al-Mutawakel, Ahmed Hamoud Ahmed Al Arami

**Affiliations:** ^1^ Department of Pharmaceutical Medicinal and Organic Chemistry, Faculty of Pharmacy, Sana’a University, Sana’a, Yemen, su.edu.ye; ^2^ Department of Pharmacology, Faculty of Pharmacy, Sana’a University, Sana’a, Yemen, su.edu.ye; ^3^ Department of Pharmaceutical, Faculty of Pharmacy, Sana’a University, Sana’a, Yemen, su.edu.ye; ^4^ Department of Pharmacy, Faculty of Medical Sciences, Al-Nasser University, Sana’a, Yemen; ^5^ Department of Pharmacy, Faculty of Medical Sciences, Saba University, Sana’a, Yemen

**Keywords:** β-lactam antibiotics, antibiotic resistance, meclizine, quality control, repurposing approach

## Abstract

Cephalosporin is one of the antibiotics that are widely used because of its broad spectrum and high effectiveness. The aim of the present research was to formulate and evaluate the antimicrobial effect of meclizine (ME) alone and in combination with cefixime (CFM) and cefuroxime (CXM). Evaluation of antibacterial activities was determined by using *Klebsiella* (sensitive and ESBL), *Escherichia coli, Staphylococcus aureus* (sensitive and MRSA), and *Pseudomonas aeruginosa.* The molecular docking was conducted for ME and CFM with the crystal structure of beta‐ketoacyl‐acp synthase III + malonyl/CoA. The results show that ME has the highest minimum inhibitory concentrations (MICs) activity against *sensitive Klebsiella* spp., followed by *sensitive S. aureus*. The combination of ME and CXM demonstrated a synergistic activity against both sensitive and resistant *S. aureus* and *sensitive Klebsiella* with an interpretation of minimum bactericidal concentration (MBC) of < 0.06, 0.1875, and 0.33, respectively. Meanwhile, partial synergism activity is observed against *E. coli*, and indifference activity against *P. aeruginosa* and *Klebsiella* ESBL with an interpretation FBC of 0.75, 1.2, and 1.25, respectively. The ME and CFM combination showed partial synergism activity against sensitive *Klebsiella, E. coli,* and *P. aeruginosa* with an interpretation FBC of 0.625, 0.75, and 0.75, respectively. While indifference activity was recorded against both sensitive and MRSA *S. aureus* and *Klebsiella* ESBL with interpretation FBC of 4, 1.25, and 1.5, respectively. The ME and CXM combination showed the highest activity against Gram‐positive bacteria and *sensitive Klebsiella*, while the ME and CFM combination showed the highest activity against Gram‐negative bacteria. This combination may consider be a promising formula as an antimicrobial drug.

## 1. Introduction

**Figure 1:**
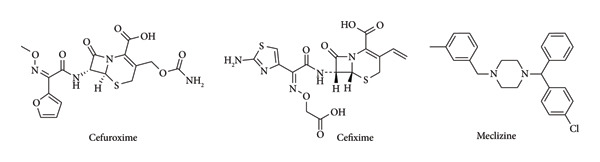
Chemical structures of cefuroxime, cefixime, and meclizine.

Over the last few decades, high multidrug resistance rates have dramatically increased because of the inappropriate initial therapy of antibiotics, which in turn has become a serious problem for health care centers worldwide. In 2014, de Kraker et al. published a review that estimated that antimicrobial resistance (AMR) could lead to 10 million deaths each year by 2050 [1]. The growing number of resistant pathogenic bacteria (such as *Escherichia coli, Staphylococcus aureus, Mycobacterium tuberculosis, Neisseria gonorrhoeae,* and vancomycin*-*resistant enterococci) gives an alarm to the worldwide community. The increase of AMR leads to the reduction of usable therapeutic agents and prolongs the length of hospital stays due to the absence of effective oral antibiotics. Therefore, searching for novel antimicrobial agents with different mechanisms of action has been carried out. Antimicrobial combination therapy is an effective strategy to fight multidrug‐resistant (MDR) Gram‐negative organisms (*Pseudomonas aeruginosa, Acinetobacter baumannii*, and Enterobacteriaceae), which have emerged as a major threat to hospitalized patients with mortality rates ranging from 30% to 70%. Combined antibiotic therapy has a broader antibacterial spectrum, can shorten the length of therapy and reduce the dose of medicine, and thus enhance the synergistic effect and reduce the risk of emerging resistance during therapy. The World Health Organization (WHO), however, has recommended dual antibiotic combinations for the AMR against several bacteria, including *Neisseria gonorrhoeae* [2]. Cefotaxime and cefixime (CFM) are β‐lactam antibiotics (Figure 1) with broad‐spectrum bactericidal activity against both Gram‐positive and Gram‐negative bacteria. Its bactericidal activity results from the inhibition of cell wall synthesis via affinity for penicillin‐binding proteins (PBPs). PBPs are targets for β‐lactam antibiotics [3, 4]. These antibiotics have been combined with different drugs as AMR drugs. Mei Huang et al. established a synergistic antibacterial effect when cefuroxime (CXM) was combined with dihydroartemisinin (DHA) against *Escherichia coli* with a fractional inhibition concentration index (FICI) of 0.375 [5]. Additionally, CXM has been combined with nonsteroidal anti‐inflammatory drugs (NSAIDs) ibuprofen/aspirin and shown antibacterial activity with a synergistic effect against methicillin‐resistant *Staphylococcus aureus* with FICI of 0.5 and 0.28, respectively [6]. On the other hand, Rawat et al. reported a superiority in activity of CFM–clavulanic acid over amoxycillin–clavulanic acid in the treatment of infections due to ESBL‐producing *E. coli* and *K. pneumoniae* [7]. Y.D. Bakthavatchalam et al. demonstrated a synergistic effect of the CFM–ofloxacin combination against *Salmonella Typhi*. This activity is due to the CFM, which inhibits cell wall synthesis, and ofloxacin, which affects the DNA gyrase activity [8]. The presence of the piperazine moiety in ofloxacin and other drugs such as ciprofloxacin and levofloxacin boosts the antimicrobial activity due to its large polar surface area, structural rigidity, and hydrogen‐bond acceptors and donors [9]. Prior in silico work by Al‐Madhagi et al. demonstrated that ME has potential biological activity beyond its antihistaminic role, including inhibitory effects on microbial targets through stable binding interactions [10]. Our current docking study supports these findings, showing that ME binds favorably to the active site of beta‐ketoacyl‐ACP synthase III, suggesting a plausible mechanism for its antibacterial activity [10].

The rising prevalence of MDR and extensively drug‐resistant (XDR) bacterial pathogens represents a serious and escalating threat to global public health. Traditional antibiotic development pipelines have become increasingly limited due to scientific, regulatory, and economic challenges. In this context, drug repurposing—the strategy of finding new antimicrobial uses for existing, approved drugs—offers a compelling and efficient alternative. By building on established safety and pharmacokinetic profiles, drug repurposing can significantly accelerate the discovery and deployment of effective treatment strategies against resistant infections [11]. Therefore, based on the previous literature review, the objectives of the present research are to: (a) investigate the antimicrobial activity of ME alone and in combination with CFM and CXM as cephalosporin antibiotics against specific Gram‐positive and Gram‐negative bacteria; (b) determine minimal inhibition concentration (MIC) and minimal bactericidal concentration (MBC) as well as evaluate the synergism, addition, and antagonistic effects of ME and its active combination against the selected bacteria; and (c) formulate oral tablets of ME dual antibiotics. To the best of our knowledge, there is no antimicrobial study of ME with cephalosporin antibiotics.

## 2. Experiments and Methods

### 2.1. Reagents and Materials

For antimicrobial analysis, CFM and ME were purchased as raw materials from Global Pharma Industry, while CXM axetil as an injection was purchased from Sebla (India). Controlled test desks were obtained from the blood bank in Sana’a. Ethanol 96% and dimethylsulfoxide 99% were purchased and used as received without further purification. All chemicals used were of the analytical grade evaluation and solubilized in distilled water (CXM), ethanol 96% (ME) and dimethylsulfoxide 99% (CFM). For the tablet formulation, CFM, CXM axetil, and ME were purchased as raw materials from Global Pharma Industry. Avicel 101, Avicel 102, crystalline sorbitol, lactose anhydrous, HPMC, granulated mannitol, crospovidone, magnesium stearate, aerosil, povidone, croscarmellose, sodium lauryl sulfate (SLS), silica gel, methanol, hydrochloric acid, and acetonitrile were obtained as a gift from Shaphaco Industry, Yedco Industry, and Pharmacare Industry.

### 2.2. Instruments

Electronic balance: model: ME 204; serial no.: B801229195, Tablet Hardness Tester PTB 111 (Compbell Electronics, Germany), Disintegration Tester DIST 3 (Electrolab, Germany), Dissolution tester: model: PTWS1220; serial no.: 24150, Friabilator, HLPC‐size: LC Column 250 × 4.6 mm Desc: kinetex ∗ 5 μm C18 100A (Technochrom, Spanish), HPLC‐PUMP: Waters 1525, PDA Detector: Waters 32998, Ultrasonic Bath/Cleaner: model: SCT‐sonic6; serial no.: SCT‐sonic6P391077, and 3520 pH Meter (Jenway, United Kingdom).

### 2.3. Microorganisms

Two isolated (sensitive *E. coli* and *Klebsiella* spp. species) and four standard bacteria (sensitive *P. aeruginosa*, *S. aureus* MRSA ATCC, and *Klebsiella* spp. ESBL) were obtained from the National Center of Public Health laboratory, Sana’a, Yemen.

#### 2.3.1. Ethical Handling of Bacterial Strains

All bacterial strains used in this study were obtained from the National Center of Public Health Laboratory, Sana’a, Yemen, with institutional permission. The strains were handled strictly according to biosafety guidelines and standard laboratory practices to ensure ethical and safe conduct of microbiological procedures.

### 2.4. Methodology of Antimicrobial Tests

#### 2.4.1. Identification of Bacteria

The strains were further identified by using chocolate agar and MacConkey methods [12]. Examination of bacteria microscopically was also carried out using crystal violet stain (blue or purple), iodine stain as a mordant, acetone–alcohol decolorization, and safranin or natural red stain tests [13].

#### 2.4.2. Biochemical Tests

The catalase test, coagulase test, oxidase test, Kligler iron agar, sulphide indole motility medium (SIM), citrate utilization test, and urease test were carried out, and the details are represented in Supporting Section 1 [14, 15].

#### 2.4.3. Antimicrobial Susceptibility Tests

The antimicrobial susceptibility testing was carried out by using Mueller–Hinton agar, and the obtained results were summarized in Table 1 and then compared with zone size in the interpretative chart [5, 8]. Mueller–Hinton agar was prepared by suspending 38 g of Mueller–Hinton agar dehydrated powder in 1 L of distilled/deionized water. The mixture was mixed until completely dissolved and then gently heated, sterilized by autoclaving at 121°C for 15 min, and finally the media was poured into the petri dish and left to solidify. To confirm MRSA strains, the cefoxitin diffusion disc method was carried out on Mueller–Hinton agar by using a 30 μg cefoxitin disc. The inhibition zone diameter of 21 mm was reported as methicillin‐resistant, whilst the diameter of 22 mm was considered as methicillin‐sensitive [16]. *Klebsiella* strains were screened as ESBL screening‐positive on the basis of resistance to ceftazidime. The screen‐positive *K. pneumoniae* strains were further processed for the double disc synergy test (DDST). In DDST, a disc of amoxicillin–clavulanic acid was placed in the center of a Mueller–Hinton agar plate at a distance of 20 mm from ceftazidime, cefotaxime, and ceftriaxone.

**Table 1:** Inhibition zones (mm) of interpretative standards for positive and negative bacteria.

Type of antibacterial	Type of bacteria					
	*S*.*A* *u* *r* *e* *u* *s* ^1^	*M* *R* *S* *A* ^2^	*E*.*c* *o* *l* *i* ^1^	*K* *l* *e* *b* *s* *i* *e* *l* *l* *a* ^1^	*K* *l* *e* *b* *s* *i* *e* *l* *l* *a* *E* *S* *B* *L* ^3^	*P* *s* *e* *u* *d* *o* *m* *o* *n* *a* *s* ^1^ *Aeruginosa*
Penicillin 6 µg	1 ± 0.01	—	—	—	—	—
Erythromycin 15 µg	2 ± 0.01	—	—	—	—	—
Clindamycin 2 µg	25 ± 0.05	—	—	—	—	—
Cefoxitin 30 µg	23 ± 0.09	< 21	—	—	—	—
Cefuroxime 5 µg	—	—	21 ± 0.08	20 ± 0.02	—	—
Cefixime 30 µg	—	—	22 ± 0.02	22 ± 0.01	—	—
Ceftazidine 30 µg	—	—	—	—	< 10	26 ± 0.01
Meropenem 10 µg	—	—	—	—	—	30 ± 0.04
Amikacin 30 µg	—	—	—	—	—	26 ± 0.03
Gentamycin 10 mg	—	—	—	—	—	17 ± 0.04
Ciprofloxacin 5 µg	—	—	—	—	—	30 ± 0.06
Piperacillin+ Tazobactam 110 μg	—	—	—	—	—	26 ± 0.01

^1^Sensitivity of *S. aureus*, *E. coli*, and *Klebsiella* was confirmed by obtaining IZ diameters within the range of selected antibiotic discs.

^2^Cefoxitin gave an IZ below 21 mm, which proved that *S. aureus* is methicillin‐resistant.

^3^After screening of *Klebsiella* strains as ESBL screening positive on the basis of resistance to ceftazidime.

#### 2.4.4. Evaluation of Antibacterial Activities

The evaluation assays of the bacteria were carried out using the well diffusion method. Antibacterial agents that were used in this study were CXM 375 mg/mL, ME 50 mg/mL, CFM 100 mg/mL, CXM + ME 187.5 + 25 mg/mL, and CFM + ME 50 + 25 mg/mL as raw materials. Antibacterial activities were evaluated by measuring the inhibition zone diameter after the agents were incubated in the plates at 37°C for 24 h. The appearance of the white color indicated the growth area; thus, the MIC value was determined from the lowest concentration that remained colorless [17].

#### 2.4.5. Determination of MIC

A broth tube dilution method was used to determine the MICs of the antiracist agents. Mueller–Hinton broth powder (21 g) was added to distilled/deionized water, and the volume was brought to 1 L. The mixture was thoroughly mixed and gently heated and brought to a boil. After that, the mixture was distributed into a flask and sterilized by autoclaving at 121°C for 15 min. In sterile test tubes, 1 mL of broth was added, followed by the addition of 1 mL of antibiotic, and then the serial dilution was run. The content was cultured with 100 mcl of bacteria and incubated for 16–20 h at 35 ± 2°C.

#### 2.4.6. Determination of MBC

The media was prepared from nutrient agar according to the manufacturer’s instructions. The media was poured into the dishes. Bring out the tubes that incubated from MIC. A swab was taken and wiped in the dishes, then incubated for 16–20 h at 35 ± 2°C.

#### 2.4.7. Synergy Testing Methods

The goal of synergy testing is to assess the in vitro interaction of antimicrobial combinations to determine whether the effect of the two antimicrobials is greater than the sum of their individual activities [18].

##### 2.4.7.1. Checkerboard Method

The checkerboard method evaluates the activities of antimicrobial combinations that have been tested at clinically achievable concentrations with twofold serial dilutions. The assay combinations are generally designed to include antimicrobials from different classes. Generally, the data generated by the checkerboard test are analyzed in terms of the fractional inhibitory concentration index (FIC). The FIC is calculated by comparing the MIC value of each agent alone with the combination‐derived MIC. Antimicrobial combinations that result in a fourfold reduction in MIC compared with MICs of agents alone are synergistic (FIC ≤ 0.5). FICs in the range 0.5–1.0 are considered nonsynergistic or additive. FICs 1 through 4 are defined as indifferent, while those in > 4 are antagonistic. The limitations of this method are that it only tests antimicrobials for a fixed incubation period, it can require a large number of reagents and resources to test different formulations of antimicrobial combinations, and it is not able to test more than two antimicrobials simultaneously. In other words, combinations of three and four antimicrobials cannot be tested [18]. FIC values were calculated by using the following formula:

(1) ∑FIC=FICA+FICB⁣,

Where FIC_*A*_ was calculated as the MIC of drug *A* in the combination divided by the MIC of drug *A* alone, and FIC_*B*⁣_ equals the MIC of drug *B* in the combination divided by the MIC of drug *B* alone.

(2) FICI=MIC of drug A in combinationMIC of drug A+MIC of drug B in combinationMIC of drug B.

In this work, the fractional bactericidal concentration index was measured as described before but by substituting the inhibitory concentration with bactericidal concentration.

### 2.5. Molecular Docking

**Figure 2:**
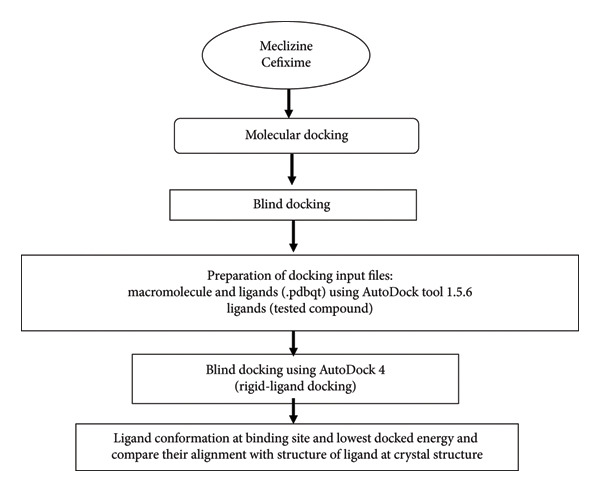
Flowchart of techniques involved in the computational study.

Molecular docking is a computational approach that examines the possibility of pose binding in a protein to a ligand and calculates the binding affinity. The molecular docking approach was used for the tested compound to find the potential protein targets used for the treatment of bacterial infection. The technique flowchart is shown in Figure 2.

#### 2.5.1. Preparation of Macromolecule Input Files for Blind Docking

The macromolecule input file for blind docking was prepared using the AutoDock tool for 1hnj (crystal structure of beta‐ketoacyl‐acp synthase lll + malonyl‐coA). Some steps involved the addition of hydrogen atoms, merging of nonpolar hydrogen atoms, assignment of Gasteiger charges and (1hnj) atom type, and checking and repairing missing atoms in proteins used for reverse docking. A grid box that defined the area of a protein involved in the docking calculation with a grid‐point spacing of 1 Å, with dimensions ranging between 126 × 126 × 126 points along the *x*, *y,* and *z*‐axes, and centered on the different proteins used, covering the whole protein using the AutoGrid tool of (1hnj). The prepared protein models were then saved in pdbqt format for docking runs.

#### 2.5.2. Preparation of Ligand Structural Files

CFM and ME were used in reverse docking, and the ChemDraw Professional 15.0 program was used to draw the two‐dimensional structure of the studied compounds. Chem3D 15.0 was used to convert the 2D structure into 3D. Energy minimization for the ligands was done using Hyperchem Pro 8.0 software, employing the steepest descent and conjugate gradient methods (termination condition set of a maximum 500 cycles or 0.1 kcal/Å mol rms gradient) [19]. The AutoDock tool 1.5.6 software was used to prepare the minimized ligand structures with the detected root of torsion and the number of torsions for flexible ligand docking to be saved as ligand pdpqt.

#### 2.5.3. Docking Protocol

The CFM, ME, and the standard ligand (flexible ligand) were docked (blind docking) using AutoDock to one target protein, pdbid 1hnj. The parameters of the flexible ligands were set to a population size of 100, and 10,000,000 energy assessments were used for 100 search runs to produce nine distinct conformations using the Lamarckian genetic algorithm search function. The resulting conformation was clustered based on the RSMD of not more than 1.5 Å. The conformation with the lowest docked energy at the binding sites in comparison to the ligand deposited in the protein crystal structure [20, 21].

### 2.6. Experimental

#### 2.6.1. Preparation of Formulations F1–F10

The formulation of all dual medications F1–F10 were described in details in the Supporting at Section 2†.

#### 2.6.2. Physiochemical Analysis

The physiochemical analysis were described in details in the supporting at Sections 3†.

### 2.7. Statistical Analysis

All assays were performed in triplicate, and results are now presented as mean ± standard deviation.

## 3. Results

### 3.1. Antibacterial Activity Study

#### 3.1.1. Antibacterial Activities of Free CXM, CFM, and ME Against Sensitive Bacteria

Table 2 summarized the antibacterial activity of CXM, CFM, and ME against sensitive Gram‐positive and Gram‐negative bacteria. The obtained MBC for CXM and CFM activity against *E. coli* was found to be 1 mg/mL with a similar range of IZ (25 and 26 mm, respectively). Besides, against *Klebsiella* spp., the inhibition zone and MBC of CXM and CFM were recorded at 42 mm, 0.375 mg/mL and 47 mm, 0.5 mg/mL, respectively.

**Table 2:** Dose, IZ, MIC, and MBC of CXM, CFM, and ME alone against sensitive positive and negative bacteria.

Type of bacteria	Bacteria	Agent	Dose (mg/mL)	IZ (mm)	MIC (mg/mL)	MBC (mg/mL)
Sensitive (Gram‐positive)	*S. aureus* ATCC	CXM	375	45 ± 0.01	0.375 ± 0.01	0.75 ± 0.01
		CFM	100	24 ± 0.02	0.375 ± 0.03	0.75 ± 0.01
		ME	50	44 ± 0.03	0.375 ± 0.05	0.75 ± 0.01

Sensitive (Gram‐negative)	*E. coli*	CXM	375	25 ± 0.06	0.125 ± 0.01	1 ± 0.01
		CFM	100	26 ± 0.07	0.5 ± 0.01	1 ± 0.01
		ME	50	N/A	0.5 ± 0.01	0.5 ± 0.01
	*Klebsiella* spp.	CXM	375	42 ± 0.08	0.1875	0.375 ± 0.01
		CFM	100	46 ± 0.06	0.25	0.5 ± 0.01
		ME	50	28 ± 0.07	0.031	0.125 ± 0.01
	*P. aeruginosa* ATCC	CXM	375	35 ± 0.05	> 7.5	> 7.5
		CFM	100	40 ± 0.05	1.875 ± 0.02	3.75 ± 0.05
		ME	50	N/A	0.9375 ± 0.01	1.875 ± 0.03

It is worthy to mention that with only high doses of CXM (375 mg/mL) and CFM (100 mg/mL), both drugs showed activity against *P. aeruginosa* ATCC with MBC = > 7.5 and 3.75 mg/mL, respectively. More importantly, ME provides an antibacterial activity against *S. aureus,* which is similar to CXM and CFM with MBC 0.75 mg/mL and IZ = 44 mm. Notably, ME shows the highest activity against *Klebsiella* spp. with IZ = 28 mm and MBC = 0.125 mg/mL, followed by *E. coli* and *P. aeruginosa* ATCC with MBC = 0.5 mg/mL and 1.875 mg/mL, respectively.

#### 3.1.2. Antibacterial Activities of CXM, CFM, and ME Alone Against Resistant Bacteria

Table 3 illustrates the antibacterial activity of CXM, CFM, and ME against resistant Gram‐positive and Gram‐negative bacteria.

**Table 3:** Dose, IZ, MIC, and MBC of CXM, CFM, and ME alone against sensitive standard positive and negative bacteria.

Type of bacteria	Bacteria	Agent	Dose (mg/mL)	IZ (mm)	MIC (mg/mL)	MBC (mg/mL)
Resistance (Gram‐positive)	*S. aureus* MRSA ATCC	CXM	375	36 ± 0.06	0.75 ± 0.01	1.5 ± 0.01
		CFM	100	—	0.75 ± 0.01	3 ± 0.01
		ME	50	15 ± 0.05	0.75 ± 0.01	0.75 ± 0.01

Resistance (Gram‐negative)	*Klebsiella* spp. ESBL	CXM	375	30 ± 0.06	2.8125 ± 0.01	3.75 ± 0.06
		CFM	100	—	1.125 ± 0.01	1.5 ± 0.01
		ME	50	30 ± 0.04	0.5625 ± 0.01	0.75 ± 0.01

For the conventional antibiotics CXM and CFM, the observed MIC was similar for both at 0.75 mg/mL against *S. aureus* MRSA ATCC. Whereas, toward *Klebsiella* spp. ESBL, the MIC was observed at 2.8125 mg/mL and 1.125 mg/mL for CXM and CFM, respectively. Significant activities were observed with ME against *S. aureus* MRSA ATCC and *Klebsiella* spp. ESBL with MIC 0.75 mg/mL and 0.5625 mg/mL, respectively.

The differences in antibacterial efficacy were statistically significant, as confirmed by two‐way ANOVA (*p* < 0.05), indicating a meaningful variation in the performance of the agents across bacterial strains, as summarized in Table 4.

**Table 4:** Statistical analysis using two‐way ANOVA without replication for sensitive and resistant species.

Source of variation	SS	df	MS	*F*	*p*‐value	*F* crit
*ANOVA—S. aureus (sensitive and resistant) species*						
Rows	20366.02	4	5091.506	1.104074	0.398806	3.259167
Columns	61667.41	3	20555.8	4.457447	0.025281	3.490295
Error	55338.77	12	4611.564			
Total	137372.2	19				

*ANOVA—E. coli, Klebsiella, and P. aeruginosa (sensitive and resistant species)*						
Rows	58428.83	10	5842.883	1.20042	0.329723	2.16458
Columns	182980.2	3	60993.41	12.5311	1.75E − 05	2.922277
Error	146020.9	30	4867.364			
Total	387430	43				

#### 3.1.3. Antibacterial Activities of ME and CXM Combination Against Sensitive and Resistant Gram‐Positive and Gram‐Negative Bacteria

In this study, the MBCs of the ME and CXM combination against all strains were significantly lower than those of ME and CXM alone, as shown in Table 4. The MBC of free ME and CXM for the studied bacteria ranged from 0.125 to > 7.5 g/mL compared to 1.875 to < 0.0234375 g/mL for ME and CXM antibiotic combinations. Additionally, for the sensitive and resistant *S. aureus* as positive bacteria, the ∑FBC value of ME and CXM was found to be < 0.0625 and 0.1875, respectively.

Moreover, among the other tested pathogens, this combination showed ∑FBC = 0.33 and 0.75 against sensitive *Klebsiella* spp. and sensitive *E. coli,* respectively. Meanwhile, against sensitive *Klebsiella* spp., ESBI and *P. aeruginosa,* this combination showed ∑FBC = 1.2 and 1.25, respectively (Table 5).

**Table 5:** Antibacterial activity of CXM and ME combination against positive and negative bacteria.

Bacteria	Agent	Dose (mg/mL)	IZ (mm)	MIC (mg/mL)	MBC (mg/mL)	FBC value	∑FBC	Interpretation FBC
*Negative*								
*Klebsiella* sensitive	CXM	187.5	51	0.002	0.75 ± 0.01	0.0833	0.33	S
	ME	25			0.75 ± 0.01	0.25		

*E. coli* sensitive	CXM	187.5	24	0.062	0.1875 ± 0.02	0.25	0.75	PS
	ME	25			0.1875 ± 0.04	0.5		

*Klebsiella* spp. ESBI	CXM	187.5	44	0.002	0.75 ± 0.01	0.2	1.2	In
	ME	25			0.75 ± 0.01	1		

*P. aeruginosa sensitive*	CXM	187.5	52	0.9375	1.875 ± 0.02	< 0.25	1.25	In
	ME	25			1.875 ± 0.03	1		

*Positive*								
*S. aureus* sensitive	CXM	187.5	50	< 0.02344	< 0.02348	< 0.03125	< 0.06	S
	ME	25			< 0.02348	< 0.03125		

*S. aureus* MRSA	CXM	187.5	44	0.04687	0.09375	0.0625	0.1875	S
	ME	25			0.09375	0.125		

*Note:* S: synergism; In: indifference.

Abbreviations: FBC, fractional bactericidal concentration; IZ, inhibition zone; MBC, minimum bactericidal concentration; MIC, minimum inhibitory concentration; PS, partial synergism.

#### 3.1.4. Antibacterial Activities of ME and CFM Combination Against Sensitive and Resistant Gram‐Positive and Gram‐Negative Bacteria

Antibacterial activities of the ME and CFM combination against sensitive and resistant Gram‐positive and Gram‐negative bacteria were analyzed by measuring IZ, MIC, MBC, and FBC, and the results were tabulated in Table 6. As can be seen from Table 5, the MICs and MBCs of the ME and CFM combination were less than those of free CFM and ME.

**Table 6:** Antibacterial activity of CFM and ME combination against positive and negative bacteria.

Bacteria	Agent	Dose (mg/mL)	IZ (mm)	MIC (mg/mL)	MBC (mg/mL)	FBC value	∑FBC	Interpretation FBC
*Negative*								
*E. coli* sensitive	CFM	50	23	0.062 ± 0.01	0.25 ± 0.01	0.25	0.75	PS
	ME	25			0.25 ± 0.01	0.5		

*Klebsiella* spp. sensitive	CFM	50	59	0.5625 ± 0.01	0.0625 ± 0.01	0.125	0.625	PS
	ME	25			0.0625 ± 0.01	0.5		

*P. aeruginosa* sensitive	CFM	187.5	25	0.4687 ± 0.01	0.9375 ± 0.01	0.25	0.75	PS
	ME	25			0.9375 ± 0.01	0.5		

*Klebsiella* spp. ESBI	CFM	50	35	0.5625 ± 0.01	0.75 ± 0.01	0.5	1.5	In
	ME	25			0.75 ± 0.01	1		

*Positive*								
*S. aureus* sensitive	CFM	50	44	1.5 ± 0.01	1.5 ± 0.01	2	4	In
	ME	25			1.5 ± 0.01	2		

*S. aureus* MRSA	CFM	50	—	0.375 ± 0.01	0.75 ± 0.01	0.25	1.25	In
	ME	25			0.75 ± 0.01	1		

*Note:* S: synergism; In: indifference.

Abbreviations: FBC, fractional bactericidal concentration; IZ, inhibition zone; MBC, minimum bactericidal concentration; MIC, minimum inhibitory concentration; PS, partial synergism.

In this study, the finding established good to moderate antibacterial activity against the negative bacteria with MIC ranging between 0.031 mg/mL and 0.5625 mg/mL and against positive bacteria with MIC = 0.375–1.5 mg/mL compared with CFM. In contrast, the ∑FBC that was recorded against sensitive *Klebsiella* spp. ESBI*, S. aureus*, and MRSA *S. aureus* was 1.4, 4, and 1.25, respectively.

### 3.2. In Silico Study

The PASS tool is a crucial method to predict the possible activity scores of the drug for different cell screens. However, the PASS results show that ME exhibits a cephalosporin‐C deacetylase inhibitor with a possible activity value of 67.7%. While investigating the molecular docking, the ligand interaction between ME associated with 1hnj shows different hydrophobic intermolecular interactions (Figure 3). In particular, alkyl and π–alkyl stacked bonds with four proteins: one at ILE156, two at ALA246, one at VAL212, and one at CYS112 were observed. Additionally, one π–sigma bond binding with LEU189 and one π–sulfur bond binding with MET207 were also observed (Figure 3).

Figure 3(a and b) The hydrophobicity interaction between ME within the binding site of the 1hnj crystal structure inhibitor; (c) H‐bonding of ME with 1hni; (d) schematic representation of binding interaction of ME with 1hnj.

**Figure:**
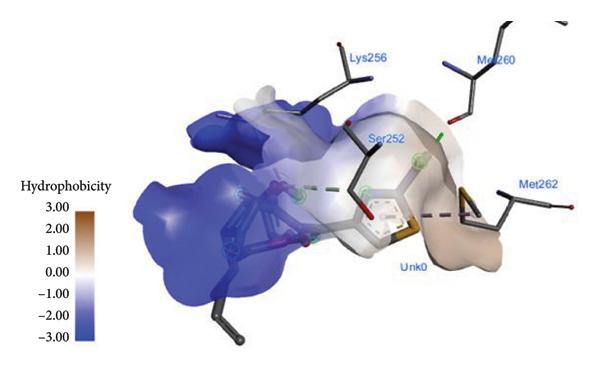
(a)

**Figure:**
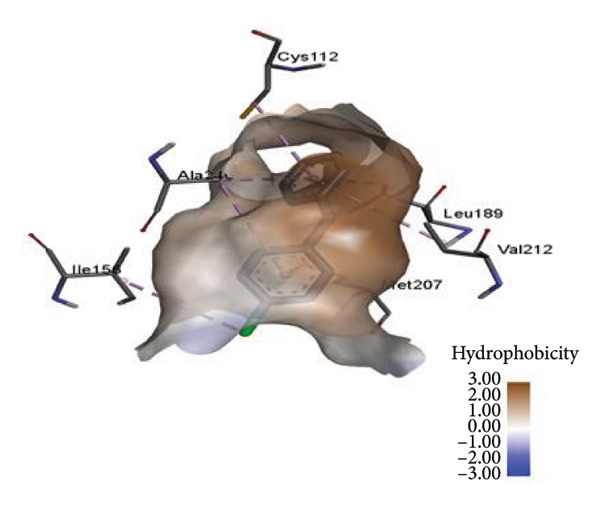
(b)

**Figure:**
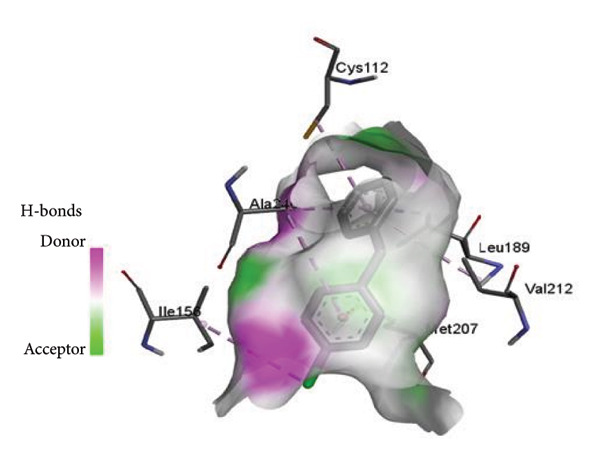
(c)

**Figure:**
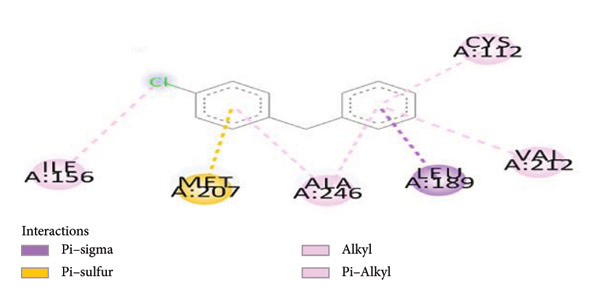
(d)

**Figure 4:**
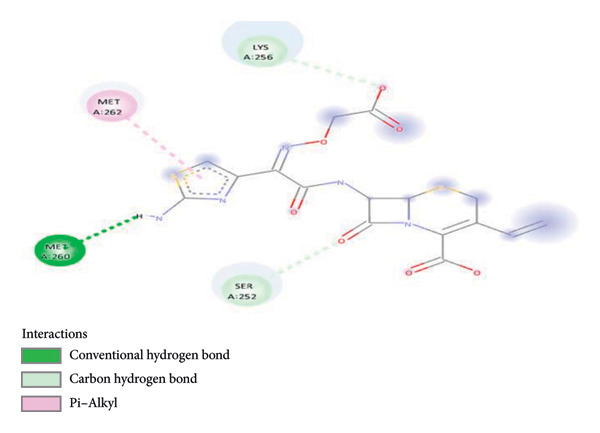
Schematic representation of binding interaction of CFM with 1hnj crystal structure inhibitor.

On the other hand, the binding of CFM within the binding protein 1hnj crystal structure inhibitor was observed at four binding proteins: one at the π–alkyl bond binding with MET262, one at the conventional hydrogen bond with MET260, and two conventional carbon–hydrogen bond bindings with SER252 and LYS256 (Figure 4).

### 3.3. Formulation of ME + CXM and ME + CFM Dual Antibiotics

Ten formulations based on ME with CXM and CFM were prepared in different manners, as shown in Table 7. Physicochemical evaluation of the dual antibiotics (ME + CXM and ME + CFM) was carried out based on weight variation, hardness, friability, and disintegration, and the results are tabulated in Table 8. Further, the dissolution test was also conducted to confirm the absorption and bioavailability of the formulations, and the results are presented in Table 8.

**Table 7:** Materials and quantities that were used to formulate the dual antibiotic combinations CXM and CFM with ME.

Material	F1	F2	F3	F4	F5	F6	F7	F8	F9	F10
CFM trihydrate	113.48	113.48	—	—	—	113.48	112	—	112	—
CXM axetil	—	—	240.57	240.57	240.57	—	—	240.57	—	240.57
ME HCl	25	25	25	25	25	25	25	25	25	25
Avicel 101	—	102.52	—	—	75.43	—	100	101.43	100	101.43
Avicel 102	—	—	—	93.43	—	150.52	—	—	—	—
Granulated mannitol	—	65	—	—	50	—	—	—	—	—
Crystalline sorbitol	—	—	114.93	—	—	—	—	—	—	—
Lactose‐anhydrous	192.02	—	—	—	—	—	—	—	—	—
Crospovidone	25	20	25	—	27	16	80	80	85	85
Povidone	—	15	—	—	18	—	—	—	—	—
Croscarmellose	—	—	—	27	—	—	40	40	45	45
HPMC	40	—	40	—	—	—	—	—	—	—
SLS	—	—	—	5	5	—	10	10	—	—
Aerosil	—	4.5	—	4.5	4.5	4	5	5	5	5
Mg stearate	4.5	4.5	4.5	4.5	4.5	6	5	5	5	5
Sodium carbonate	—	—	—	—	—	—	—	—	100	—
Total (mg)	400	400	450	400	450	315	377	507	477	507

**Table 8:** Physicochemical properties (average weight, friability, hardness, and disintegration) for the tablets containing a combination of cephalosporins (CXM and CFM) with ME.

Formula	Average weight SD (mg)	Friability (%)	Average hardness (kg/cm ± SD)	Disintegration rate mean ± SD (min)
F4	404.68 ± 13.12	0.823	11.76 ± 0.44	2.456 ± 0.179
F5	453.72 ± 8.93	0.109	24.26 ± 0.488	1.771 ± 0.084
F6	316.5 ± 7.37	0.148	38.96 ± 2.05	2.675 ± 0.236
F7	376.73 ± 5.06	0.055	20.204 ± 3.05	1.08 ± 0.083
F8	512.15 ± 6.12	0.111	12.234 ± 2.11	1.006 ± 0.084
F9	479.05 ± 6.003	0.222	12.254 ± 0.987	0.5 ± 0.003
F10	514.9 ± 6.884	0.306	12.217 ± 1.021	0.511 ± 0.002

According to the physicochemical evaluation, only the F4–F10 formulations fall within the acceptable weight variation range of 10% as specified in the USP. Although, the failing formulation F1–F3 may have contributed to the incompatible chemical storage and contamination. The weight variation of F4–F10 formulations ranged from 316.5 ± 7.37 mg to 514.9 ± 6.884 mg. Therefore, these formulations were selected for further analysis. In the friability test, the friability values of formulations F4–F10 ranged from 0.055% to 0.823%. For the hardness test, the study revealed that the average compression force recorded was in the range of 11.76 ± 0.44 to 38.96 ± 2.05 N, which is less than the minimum value of 50 N. Further, the mean disintegration rate was recorded from 0.5 ± 0.003 min to 2.675 ± 0.236 min (Table 8). In terms of the dissolution rate, F9 and F10 display the highest drug release (Table 9). In particular, formulation F9 showed drug release of 102.6% and 98.3% for CFM and ME, respectively, while the dissolution rate of F10 was recorded as 101.38% and 99.4% for CXM and ME, respectively.

**Table 9:** Results of the dissolution rate for the selected formulations F4–F10.

Formula	Mean results (%)			Remark
	CFM	CXM	ME	
F4	—	65	55	Rejected
F5	—	70	65	Rejected
F6	100.3	—	56	Rejected
F7	102.2	—	60.3	Rejected
F8	—	100.4	88	Rejected
F9	102.6	—	98.3	Acceptance
F10	—	101.38	99.4	Acceptance

## 4. Discussion

The antibacterial activities were tested for free ME and its combination with CXM and CFM against the Gram‐positive and Gram‐negative bacteria. The selected bacteria were as follows: *Staphylococcus aureus* (*S. aureus*) (sensitive and MRSA), ATCC *Pseudomonas aeruginosa* (*P. aeruginosa*)*, Klebsiella* spp. (sensitive and ESBL), and *E. coli.* The MIC of ME and its combination with CXM and CFM was determined by using the tube dilution method, and the combinational effect was performed using the checkerboard assay.

In terms of antibacterial activity, ME shows the highest activity against Gram‐positive bacteria compared to CXM and CFM. The ME activity can be attributed to its poor water solubility, which enables it to penetrate the cell wall of Gram‐positive bacteria. In this study, the MBCs of the ME and CXM combination against all strains were significantly lower than those of the ME and CXM alone (Table 4). This indicates the new drug formulations have a greater effect on bacteria compared with free antibiotics. According to the MIC values, it was noticed that the antibiotic combination exhibited a 16‐fold increase in potency compared to CXM against sensitive and resistant *S. aureus* ATCC bacteria. Further, the strongest antimicrobial effect of the ME and CXM combination is observed against sensitive *Klebsiella* spp., which is 94‐fold stronger than CXM and 16‐fold higher than ME. Also, this combination displayed a twofold higher MIC as compared with CXM against sensitive *E. coli*. The antibiotic combinations ME and CFM displayed antimicrobial activity with a partial synergism effect for sensitive *E. coli*, *Klebsiella* spp., and *P. aeruginosa* with ∑FBC values of 0.75, 0.625, and 0.75, respectively. It can be inferred the MICs of this combination exhibit eightfold higher antimicrobial activity against *E. coli* and *Klebsiella* spp. and fourfold increases against *P. aeruginosa* in comparison to CFM. In contrast, an indifference effect was observed against sensitive *Klebsiella* spp. ESBI*, S. aureus*, and MRSA *S. aureus* with ∑FBC = 1.4, 4, and 1.25, respectively.

In this study, the MBCs of the ME and CXM combination against all strains were significantly lower than those of ME and CXM alone, as shown in Table 4. This indicates the new drug formulations have a greater effect on bacteria compared with free antibiotics. The antibiotic combinations ME and CXM demonstrated promising antimicrobial activity with a synergism effect for both sensitive and resistant positive bacteria (*S. aureus*) with ∑FBC value = < 0.0625 and 0.1875, respectively. Moreover, among the other tested pathogens, this combination showed synergism and partial synergism effects against sensitive *Klebsiella* spp. and sensitive *E. coli* with ∑FBC = 0.33 and 0.75, respectively. Meanwhile, an indifference effect was observed against sensitive *Klebsiella* spp. ESBI and *P. aeruginosa* with ∑FBC = 1.2 and 1.25, respectively (Table 4).

In this study, in silico methods were employed to identify potential binding sites and to predict the mechanism of protein–ligand interactions between the target protein 1hnj (beta‐ketoacyl‐acyl carrier protein synthase III) and the compounds ME and CFMCFM. The selected protein plays a critical role in bacterial fatty acid biosynthesis, making it a relevant antibacterial target.

Molecular docking analysis using AutoDock revealed that ME exhibited a binding energy of –2.79 kcal/mol with an estimated inhibitory constant of 8.95 mM, indicating spontaneous binding. This is consistent with literature noting that negative binding energy values reflect favorable, energy‐independent binding processes [22]. In comparison, CFM showed a weaker interaction, with a maximum binding energy of –1.26 kcal/mol. The docking protocol utilized in this study was based on the AutoDock algorithm developed by Trott and Olson, which has been widely validated for its improved speed and accuracy in predicting ligand‐binding conformations [20]. The low binding energy observed for ME in our results aligns with this tool’s predictive capabilities and supports the hypothesis of favorable ligand–protein interaction.

The physicochemical evaluation contributed to the study of the dual drug combination stability, and the investigation showed that only the F4–F10 formulations fall within the acceptable weight variation range of 10% as specified in the USP. The failing formulation F1–F3 may have contributed to the incompatible chemical storage and contamination. In the friability test, the friability values of formulations F4–F10 ranged from 0.055% to 0.823%. These formulations have met the specification of the USP, which specifies that friability tests must not lose more than 1% of their initial weight. Furthermore, the hardness and disintegration tests are connected together, in that as the hardness value is increased, the disintegration time is increased and vice versa. Additionally, the dissolution rate of drugs should be limited to 90%–100% as stipulated by USP. F9 and F10 display the highest drug release (Table 8). Formulation F9 showed drug release of 102.6% and 98.3% for CFM and ME, respectively. F10 showed 101.38% and 99.4% of CXM and ME, respectively. Meanwhile, the release rate of formulations F4–F8 failed to meet the USP (88%–102.6%). Drugs with poor dissolution profiles will not be sufficiently available in the body to create the preferred therapeutic effect. Therefore, based on the physicochemical evaluation, we can say that F9 and F10 show a satisfactory quality control limit for the pharmacopeia.

**Figure 5:**
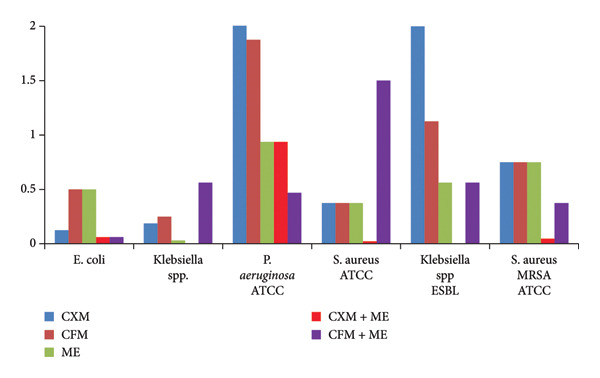
Plot of MICs versus sensitive and resistant bacteria for ME alone and the CXM and CFM combination.

A comparative trial between ME with either CXM or CFM has also been investigated and plotted in Figure 5. It can be excluded from Figure 5 that ME displays significant antibacterial activity against *P. aeruginosa* and *Klebsiella* spp. (sensitive and resistant) with MIC ranging between 0.031 and 0.937 mg/mL as compared with CXM and CFM (0.187–7.5 and 0.25–1.875, respectively). By referring to Figure 5, the dual antibiotic combination of ME and CXM is more effective against negative and positive bacteria as compared with monotherapy of ME and CXM alone along with all studied bacteria. Furthermore, ME and CFM combinations exhibit moderate activity toward the selected bacteria. It is worthy to mention that the ME and CXM combination does not show any antagonism, which indicates their potential clinical efficacy. Meanwhile, WHO documented that CFM + azithromycin and ceftriaxone + azithromycin combinations having no antagonistic interaction indicates their continuing clinical utility [23].

Our findings align with previous research demonstrating the potential of cephalosporin‐based combination therapies. For example, Huang et al. (2020) reported a synergistic effect of CXM combined with DHA against *E. coli*, showing an FICI of 0.375, which is comparable to the FICI values observed in our ME + CXM combination against sensitive *E. coli* and *Klebsiella* spp. Similarly, Chan et al. (2017) demonstrated that CXM combined with NSAIDs exhibited synergism against MRSA, consistent with our results showing a strong synergistic effect of ME + CXM against both sensitive and MRSA *S. aureus*.

In contrast, the partial synergy or indifference observed in our ME + CFM combination reflects similar variability reported in earlier studies on CFM combinations. For instance, Bakthavatchalam et al. (2017) noted moderate to partial synergistic effects of CFM–ofloxacin against *Salmonella Typhi*, supporting the notion that the interaction outcome is highly strain‐ and partner‐dependent. Our findings thus expand on the existing evidence and suggest that ME, when paired with CXM, holds greater promise in targeting both Gram‐positive and Gram‐negative resistant strains.

## 5. Conclusion

In this article, we reported that ME is a promising antibiotic drug against positive and negative bacteria (*S. aureus*, *Klebsiella* spp.*, E. coli, P. aeruginosa, Klebsiella* ESBL, and *S. aureus* MRSA). The in silico study proved that ME has good binding affinity to the protein pdb id 1hnj (crystal structure of beta‐ketoacyl‐acp synthase III + malonyl‐CoA). Two antibiotic combinations, namely ME + CXM and ME + CFM, exhibit selectivity toward positive and negative sensitive and resistant strains. The finding of this study revealed a synergistic and indifference effect with the ME + CXM combination without any antagonism effect. However, ME + CFM show partial synergistic and indifference effects against the studied bacteria. Among the prepared formulations, F9 and F10 showed excellent assay content and dissolution results. The outcome from this study highlights that the ME + CXM combination should be further evaluated in vitro and in vivo against different types of resistant pathogenic bacteria.

## Disclosure

All authors have read and agreed to the published version of the manuscript.

## Conflicts of Interest

The authors declare no conflicts of interest.

## Author Contributions

Wafa M. Al‐Madhagi: supervision, conceptualization, methodology, software, validation, formal analysis, investigation, data curation, writing–original draft, and writing–review and editing. Mohammed A. Alkhawlani: methodology, validation, formal analysis, and investigation. Ahmed M. Sabati: methodology, validation, formal analysis, and investigation. Arwa Alshargabi: formal analysis, investigation, data curation, writing–original draft, and writing–review and editing. Mohammed Mostafa Al‐Mutawakel: methodology, validation, formal analysis, investigation, data curation, and writing–original draft. Ahmed Hamoud Ahmed Al Arami: methodology, validation, formal analysis, investigation, data curation, and writing–original draft.

## Funding

The authors received no specific funding for this work.

## Supporting Information

Supporting sections are classified into the following:

Section 1: Methodology of the antimicrobial activities conducted in the study.

Section 2: Methodology of the different formula trials prepared in this study.

Section 3: Quality control tests used for evaluation of the prepared formulations.

## Supporting information


**Supporting Information** Additional supporting information can be found online in the Supporting Information section.

## Data Availability

All data generated or analysed during this study are included in this published article and its supporting information files.
