# Supplementary material for: Meclizine and Cephalosporin Combination as Promising Antibiotic Drugs: Formulation, Antimicrobial, and In Silico Studies
Source: Adv Pharmacol Pharm Sci. 2025 Oct 14;2025:8972067. doi: 10.1155/adpp/8972067 (PMC12521039; doi:10.1155/adpp/8972067)
Supplement: Supplementary file 1 — Supporting Information Additional supporting information can be found online in the Supporting Information section. [file ADPP-2025-8972067-s001.docx]

**Supplementary**

**Section 1:**

**1. Antimicrobial analysis**

***1.1 Preparation of chocolate agar***

Except hemoglobin solution, All components was added to the distilled water and complete volume to 500 ml, mixed thoroughly, and gently heated until boiling. The mixture was autoclaved at 121°C for 15 min and then cooled to 45°C-50°C. Sterile hemoglobin solution (500 ml) was added, mixed thoroughly, and finally poured into sterile petri dishes.

***1.2 Preparation of macConkey agar***

49.53 grams of dehydrated medium was suspend in 1000 ml purified/distilled water and then heated until boiling until all medium is completely dissolved. The solution was sterilized by autoclaving at 121°C for 15 minutes and cooled to 45-50°C before it poured into sterile petri plates.

**1.3 Biochemical Tests**

***1.3.1 Catalase test***

Using a loop or sterile wooden stick, a small amount of bacterial colony was transferred to a surface of glass slide. A drop of 3% H_2_O_2_ was places on the slide and well mixed. Active bubbling formation is indicated to a +ve catalase *(Staphylococcus species)*, whilst, no bubbles formation is indicated to -ve. Catalase (*Streptococcus species*).

***1.3.2 Coagulase test***

A drop of physiological saline was placed on each end of a slide. Using loop, straight wire or wooden stick, portion of the isolated colony was emulsified. A drop of human plasma was add to one of the suspensions. Clumping or clot is indicated to positive coagulase (*Streptococcus Aureus*), whilst, no clumping or clot is indicated a negative coagulase (another *Staphylococcus species*).

***1.3.3 Oxidase test***

A filter paper is placed on a clean petri dish. 2 or 3 drops of oxidase reagent is added on the filter paper. Small quantity of bacteria was taken and mixed using a wooden stick. The color changes was observed within 10 seconds. Blue-purple color is indicated to positive +ve oxidase test, whilst, no blue-purple color is indicated to -ve oxidase test.

***1.3.4 Kligler iron agar***

The media are poured as slants and inoculated with a stab to the butt followed by a streak of the slant surface. Then, the bacteria are exposed to both an anaerobic environment (butt) and an aerobic one (slant). Incubate the tube for 18‐24 hours at 35 ^o^C in an incubator. Observe the color changes.

A/A: yellow slope (Acid) / yellow butt (Acid) : indicates the fermentation of lactose and possibly glucose with no protein utilization. (e.g*. E.Coli and Klebsiellaspp*).
K/K or K/no change: red-pink slope and butt indicates no fermentation of glucose or lactose and direct utilization of protein (e.g. *Pseudomonas* ).

***1.3.5 Sulphide indole motility medium (SIM)***

SIM medium was inoculated with straight wire loop by stabbing the middle of media. The medium was incubated at 37°C for 18 –24 hours. After determining motility and H_2_S production, 3-4 drops of Kovac’s Reagent was added and the results were observed.

Motility: If bacteria is motile appear turbidity (test is positive).
Indole production: red color at the surface of the medium (e.g. *Escherichia coli, and Proteus vulgaris*).
H_2_S production: positive reaction show blacking of media. Negative show no blacking.

***1.3.6 Citrate utilization Test***Simmon’s citrate agar contains sodium citrate, ammonium ion, and bromthymol blue. Sloped was inoculated with inoculating loop. Incubate at 35 ^0^C for 48 hours. Bright blue is indicated to positive citrate *(Klebsiella and citrobacter*), whilst, no change (green) is indicated to negative citrate.

***1.3.7 Urease Test***

The test organism is cultured in a medium contains urea and the phenol red indicator. If the medium becomes pink color, thus, +ve urease (*Proteus, Klebsiella*), meanwhile, no pink color is observed, the result indicated to a -ve urease.

**Section 2:**

**2. Preparation of formulations F1-F10**

***2.1. Preparation of Cefixime trihydrate + Meclizine HCl formulation F1***

Cefiximetrihydrate (113.48 mg), Meclizine HCl (25 mg), lactose anhydrous (192.02 mg), HPMC (40 mg), and crosspovidone (25 mg) were mixed by geometric mixing for a minute and then sieved by using sieve 40. Finally, 4.5 mg of magnesium stearate was added and mixed for 2-5 seconds before it compressed.

***2.2. Preparation Cefixime trihydrate + Meclizine HCl formulation F2***

65 mg of granulated mannitol was mixed with distilled water until a paste was formed. After that, the mixture of Cefixime trihydrate (113.48 mg), Meclizine HCl (25 mg) , avicel 101 (102.25 mg), povidone (10 mg) and crosspovidone (13.34 mg) was granulated with mannitol paste, dried at 45 ^⁰^C, and then sieved by sieve 30. The rest of povidone (5 mg), crosspovidone (6.66 mg) and aerosil ( 4.5 mg) were mixed and added to the previous mixture. Finally, 4.5 mg of magnesium stearate was added and the mixture was compressed.

***2.3. Preparation of Cefuroxime axetil + Meclizine HCl formulation F3***

An accurate weigh of Cefuroxime axetil (240.57 mg), Meclizine HCl (25 mg), crystalline sorbitol (114.93 mg), HPMC (40 mg), and crosspovidone (25 mg) were mixed by geometric mixing for a minute and then sieved by sieve 40. Finally 4.5 mg of magnesium stearate was added to the previous mixture and mixed for 2-5 seconds before it compressed.

***2.4. Preparation of Cefuroxime axetil + Meclizine HCl formulation F4***

Cefuroxime axetil (240.57 mg), Meclizine HCl (25 mg), avicel 102 (93.43 mg), aerosol (4.5 mg), and cross-carmellose (27 mg) were mixed by the geometric mixing for a minute before it sieved by sieve 40. After that, the magnesium stearate (4.5 mg) and SLS (5 mg) were added to the previous mixture and mixed for 2-5 seconds and then compressed.

***2.5. Preparation of Cefuroxime axetil + Meclizine HCl formulation F5***

50 g of granulated mannitol was mixed with distilled water until a paste was formed. After that, Cefuroxime axetil (240.57 mg), Meclizine HCl (25 mg), avicel 101 (75.34 mg), povidone (12 mg), and crosspovidone (18 mg) were mixed together and granulated by mannitol paste, dried at 45^∘^C and then sieved by sieve 30. The rest of povidone (6 mg), crosspovidone (9 mg) and aerosil (4.5 mg) were mixed and added to the previous mixture. Finally 4.5 mg of magnesium stearate and 5 mg of SLS were added and then compressed.

***2.6. Preparation of Cefiximetrihydrate + Meclizine HCl formulation F6***

A mixture of Cefixime trihydrate (113.48 mg), Meclizine HCl (25 mg), avicel 102 (150.52 mg), aerosil (4 mg), and crosspovidone (16 mg) was mixed by geometric mixing for a minute and then sieved by sieve 40. Finally, 6 mg of magnesium stearate was added to the final mixture and mixed for 2-5 seconds before it compressed.

***2.7. Preparation of Cefiximetrihydrate + Meclizine HCl formulation F7***

Cefixime trihydrate (112 mg) Meclizine HCl (25 mg), avicel 101 (100 mg), aerosil (5 mg), cross-carmellose (40 mg), crosspovidone (80 mg), and SLS (10 mg) were mixed by geometric mixing for a minute and sieved by sieve 40. After that, 5 mg of magnesium stearate was added to the previous mixture and mixed for few seconds 2-5 seconds before it compressed.

***2.8. Preparation of Cefuroxime axetil + Meclizine HCl formulation F8***

Cefuroxime axetil (240.57 mg), Meclizine HCl (25 mg), avicel 101 (101.43 mg), aerosil (5 mg), cross-carmellose (40 mg), Crosspovidone (80 mg), SLS (10 mg) and magnesium stearate (1.67 mg) were mixed and compressed. Then, the tablets were ground and sieved by sieve 30. After that, 3.33 mg of magnesium stearate was added to the resulted powder and sieved by sieve 40. Finally, the mixture was mixed for 2-5 seconds and then compressed.

***2.9. Preparation of Cefiximetrihydrate + Meclizine HCl formulation F9***

Cefixime trihydrate (112 mg), Meclizine HCl (25 mg), avicel 101 (100 mg), sodium carbonate (100 mg), cross-carmellose (45 mg), crosspovidone (85 mg), and aerosil (5 mg) were mixed by geometric mixing for a minute and sieved by sieve 40. Then, 5 mg of magnesium stearate was added to the final mixture and mixed for 2-5 seconds before it compressed.

***2.10. Preparation of Cefuroxime axetil + Meclizine HCl formulation F10***

Cefuroxime axetil (240.57 mg), Meclizine HCl (25 mg), avicel101 (101.43 mg), cross-carmellose (45 mg), crosspovidone (85 mg), and aerosil (5 mg) were mixed by geometric mixing for a minute and sieved by sieve 40. After that, 5 mg of magnesium stearate was added to the previous mixture and mixed for 2-5 seconds and then it compressed.

**Section 3:**

**3. Physiochemical analysis**

***3.1. Weight variation***

The weight of tablets is carried out to ensure the proper amount of effective drug amount in the tablets. Twenty tablets were taken from each formula and weighted individually by an analytical weighting balance.

***3.2. Hardness test***

A hardness tester was used for 6 tablets which taken randomly from each formula.

***3.3. Friability***

Six tablets were taken randomly and placed on a sieve to remove the dust from the tablets with aids of a soft brush. The tablets were weighed accurately (initial weight) and placed into the drum and then rotated at a speed of 25 rpm for 4 minutes. Finally, the tablets were reweighed (final weights) to find the % weight loss. The maximum weight loss should be not more than 1% generally to be acceptable as pharmaceutical product. The percentage of friability was calculated using the following formula:

$$Friability \%= \frac{initial weight-final weight}{initial weight} \times100$$

***3.4. Disintegration test***

Six tablets from each of formula were employed for this test in prepared medium, distilled water at 37C° using the USP disintegration apparatus. The disintegration time was taken to be the time no particle remained on the basket of the system.

***3.5. Dissolution test***

***3.5.1. Dissolution of cefixime and meclizine***

The dissolution test was performed by using dissolution tester in 900 ml of 0.01 M HCl as the dissolution medium at the rotation speed 100 rpm (revolution per minute) for 60 minutes. In 100 ml volumetric flask, weight 25 mg of meclizine HCl and 112 mg of cefixime which equivalent to 100 mg of anhydrous cefixime. The mixture was dissolved in10 ml of methanol and then the volume was completed to mark with 0.01 M HCl solution. 5 ml of the solution was transferred into 50 ml volumetric flask and completed the volume with 0.01 M HCl solution. Finally, one tablet was added in each vessels and filtered through microfiltration membranes (0.45 µg). The solution was assayed by using Uv-Vis spectrophotometer at λ= 230 nm and the absorbance was recorded.

***3.5.2. Dissolution of cefuroxime and meclizine***

The dissolution test was performed by using dissolution tester in 900 ml of 0.07 M HCl as the dissolution medium at the rotation speed 100 rpm (revolution per minute) for 45 minutes. Weight 25 mg of meclizine HCl and 240.57 mg of cefuroxime axetil (which is equivalent to 200 mg of cefuroxime) and placed in 100 ml of volumetric flask and then diluted to the mark with 0.07 M HCl. 5 ml of this solution was transferred into volumetric flask (50 ml) and diluted to the mark with 0.07 M HCl. Finally, one tablet was added in each vessels and filtered through microfiltration membranes (0.45µg). The solution was assayed by using Uv-Vis spectrophotometer at λ= 230 nm and the absorbance was recorded.

**3.6. Assay test**

***3.6.1. Assay of cefixime and meclizine***

This test was carried out by using HPLC in which the stationary phase was silica gel, while the mobile phase was contains of 550 ml of 0.01M HCl, 350 ml acetonitrile, and 100 ml methanol.

***3.6.1.1. Preparation of standard solution***

Same procedure that described in section 3.5.1. was carried out, but 10 ml was transferred to 50 ml conical flask and then diluted to the mark with 0.01 M HCl.

***3.6.1.2 Preparation of sample solution***

The tablet was crushed and weigh on equivalent amount to 25 mg meclizine and 100 mg cefixime. The powder was placed in 100 ml flask and dissolved in 10 ml methanol, and then the volume was completed with 0.01 M HCl. Finally, 10 ml of the solution was transferred into 50 ml volumetric flask and diluted to the mark with 0.1 M HCl. The solution was assayed by using Uv-Vis spectrophotometer at λ= 230 nm and the absorbance was recorded.

***3.6.2. Assay of meclizine HCl +Cefuroxime axetil***

This test was carried out by using HPLC in which the stationary phase was silica gel, while the mobile phase was contains 550 ml of 0.01M HCl and 450 ml of acetonitrile. The solution was assayed by using Uv-Vis spectrophotometer at λ= 230 nm and the absorbance was recorded. Meclizine: 25mg

***3.6.2.1. Standard solution***

25 mg of meclizine HCl was accurately weighted in 100 ml flask and then the volume completed with ethanol.

***3.6.2.2. Sample solution***

The meclizine HCl tablet was crushed and weight with an equivalent amount of 25 mg in 100 ml flask and then diluted with ethanol to the mark.

**Cefuroxime: 200 mg**

**Standard solution**

50 mg of cefuroxime axetil was weighted and placed into 100 ml flask. The powder was dissolved in 10 ml of methanol and diluted to the volume with 0.07 M HCl. Finally, 4 ml of the solution was transferred into 100 ml flask and diluted to the mark with 0.07 M HCl.

**Sample solution**

40 mg of cefuroxime axetil was weighted and placed into 100 ml flask. The powder was dissolved in 10 ml of methanol and diluted to the volume with 0.07 M HCl. Finally, 4 ml of the solution was transferred into 100 ml flask and diluted to the mark with 0.07 M HCl.
